# Improvement of Predictive Scores in Burn Medicine through Different Machine Learning Approaches

**DOI:** 10.3390/healthcare11172437

**Published:** 2023-08-31

**Authors:** Sonja Verena Schmidt, Marius Drysch, Felix Reinkemeier, Johannes Maximilian Wagner, Alexander Sogorski, Elisabete Macedo Santos, Peter Zahn, Marcus Lehnhardt, Björn Behr, German Burn Registry, Flemming Puscz, Christoph Wallner

**Affiliations:** 1 Department of Plastic Surgery, BG University Hospital Bergmannsheil, Ruhr University Bochum, 44789 Bochum, Germany; 2 Department of Anesthesiology, BG University Hospital Bergmannsheil, Ruhr University Bochum, 44789 Bochum, Germany; 3 German Society for Burn Treatment (DGV), Committee of the German Burn Registry, Luisenstrasse 58-59, 10117 Berlin, Germany

**Keywords:** burn medicine, predictive scores, ABSI, Baux, machine learning, AI

## Abstract

The mortality of severely burned patients can be predicted by multiple scores which have been created over the last decades. As the treatment of burn injuries and intensive care management have improved immensely over the last years, former prediction scores seem to be losing accuracy in predicting survival. Therefore, various modifications of existing scores have been established and innovative scores have been introduced. In this study, we used data from the German Burn Registry and analyzed them regarding patient mortality using different methods of machine learning. We used Classification and Regression Trees (CARTs), random forests, XGBoost, and logistic regression regarding predictive features for patient mortality. Analyzing the data of 1401 patients via machine learning, the factors of full-thickness burns, patient’s age, and total burned surface area could be identified as the most important features regarding the prediction of patient mortality following burn trauma. Although the different methods identified similar aspects, application of machine learning shows that more data are necessary for a valid analysis. In the future, the usage of machine learning can contribute to the development of an innovative and precise predictive score in burn medicine and even to further interpretations of relevant data regarding different forms of outcome from the German Burn registry.

## 1. Introduction

Burns are complex injuries, and especially in the case of severely burned patients in need of intensive care treatment, the mortality rate is relatively high with around 5–7% [1,2]. Over the last decades, multiple scores have been introduced and edited to predict the survival and outcome of severely burned patients. 

The Baux score for instance, which only considers two variables, was published in 1961 and is one of the oldest predictive scores for burn injuries. It sums up the patient’s age and the total body surface area burned (TBSA). It predicts the outcome of patient survival with an accuracy of around 87% in elderly patients [3]. Although its prediction rate is relatively high, it does not include relevant factors such as inhalation injury or burn depth. Therefore, multiple other scores have been developed over the course of the following years. One of the most-used scores in Europe is the Abbreviated Burn Severity Index (ABSI), which was introduced in 1982 by Tobiasen and contains age, sex, inhalation injury, full thickness burns, and total body surface area burned (TBSA) as variables to predict the survival of patients after burn injuries [4]. Other relevant scores are the FLAMES score which is a burn specific extension of the APACHE II, a score for evaluating the mortality of severely sick patients in intensive care [5], or the BEAMS score (Burn Evaluation and Mortality Study) [6], which was introduced in 2013 and is based on data from Australia and New Zealand. Despite having a variety of different scores for predicting the mortality of severely burned patients, accurate prediction is still a challenge in clinical practice and recent data have led to a steady revision of existing or the introduction of new scores.

Especially in the last decade, multiple innovations regarding the treatment of burns have revolutionized the field of burn medicine. In terms of intensive care and emergency medicine, substantial progress has led to advanced treatment of patients. Regarding these trends, predictive scores established more than 20 years ago seem questionable. Testing the accuracy of prediction of the ABSI for example, Bartels et al. could identify its predictive power as insufficient regarding different factors [7]. One issue is that the ABSI interprets the female sex as an indicator for higher mortality in severely burned patients. Meanwhile, studies have proven that gender has no impact on the outcome of burn patients [8]. Furthermore, studies regarding the outcome of trauma patients on intensive care units have shown male sex to have a negative impact [9]. Another misinterpretation that was emphasized was the linear correlation between patient’s age and mortality, for which reason the author created a new age scale for a modified version of the ABSI. Deductively, these insights also lead to the assumption that the Baux Index as a predictive score based on the patient’s age must lack in accuracy as far as predicting mortality is concerned. In 2017, Salehi et al. compared six different prediction models, identifying the ABSI score with the best prediction concerning mortality [10]. The correctly detected mortality percentage by ABSI was 67.2%, which had the highest area under the curve with 85.9. Other scores included in the study were the BOBI, rBaux, FLAMES, Ryan, and APACHE II score. Overall, in this study, surviving patients were identified as younger, had less TBSA burned, and fewer inhalation injuries than deceased patients. 

Putting these findings and the current development together, it seems obvious that prediction scores in burn medicine need further revision.

The impact of artificial intelligence (AI) in medicine over the last years is undeniable. Machine learning, as a subdiscipline of AI, uses different mathematical algorithms to identify certain data patterns leading to predictive conclusions concerning selected end points.

This study aimed to use different techniques of machine learning to analyze data from the German Burn Registry collected over the last years concerning the mortality of severely burned patients to analyze correlations between given patient characteristics (independent variables) and patient outcome (dependent variable) using different techniques of supervised machine learning. The overall goal of this study was to interpret the applicability of machine learning on prediction scores in burn medicine and to compare the different methods of machine learning regarding a possible development of a new prediction score based on machine learning.

## 2. Materials and Methods

Data were collected retrospectively from the German Burn Registry, a national platform where more than 42 parameters of burn patients treated at intensive care units are collected. The registry was established in 2014 and data for this study were taken from the years 2016 to 2021. After data cleaning, datasets of 1401 adult patients were included in this study.

The registry collects patient data such as age, sex, and trauma mechanism, and relevant factors such as temperature, TBSA, grade of burns, and inhalation injury at the time of hospitalization. Furthermore, parameters during the hospital stay such as administered volume and number and timing of surgery are documented as well as the outcome, such as mortality, duration of stay in hospital, and complications. To generate an overview of the variables considered for the different methods of machine learning, we created a table in which the variables and their means and standard errors are depicted. For feature selection, some indicators which were not measured during the time period (for example, frostbites) were erased.

For analyzing the data, different methods of machine learning were used: CAR trees, random forest, XGBoost and logistic regression.

### 2.1. Classification and Regression Trees

The Classification and Regression Trees (CARTs) are a supervised machine learning method which can be used for different kinds of data and are also known as decision trees. Data are split into several groups based on different variables which make the most homogenous groups, meaning generating the lowest entropy. After that, recursive splitting was performed, which means the splitting process was performed repeatedly (n_estimators = 1000). For the decision tree classifier (version: 1.2.2, scikit-learn), a train size of 0.8 and test size of 0.2 were chosen. A maximum depth of 2 was chosen. Data were afterwards shown in graphs created with Adobe Illustrator (Version 27.3.1, San José, CA, USA).

### 2.2. Random Forest

Random forests exist of multiple decision trees and therefore are a form of supervised machine learning as well. We used the Gini importance, a measure for impurity or entropy, as a classification criterion. For the decision tree classifier (version: 1.2.2, scikit-learn), a train size of 0.8 and test size of 0.2 were chosen. A maximum depth of 2 was chosen. After generating random forests, graphs were created with Adobe Illustrator (Version 27.3.1, San José, CA, USA).

### 2.3. XGBoost

eXtreme Gradient Boosting or XGBoost is an open-source software library that helps build a regularizing gradient framework. We trained an XGBoost algorithm in Python with patient data from the German Burn registry to generate a predictive model that detects the most predictable risk factors for mortality in burn patients treated in an intensive care unit. The aim was to generate a model that is accurate on unseen data and for predicting the mortality of a patient. The settings were chosen for the XGBoost with a learning_rate 0.1, max_depth 2, alpha = 10, and n_estimators = 1000. A train size of 0.8 and test size of 0.2 were implemented. Subsequently, k-fold cross validation was performed to ensure that the training dataset was used for training and validation. 

### 2.4. Statistical Analysis

Data were collected in Microsoft Excel (Redmond, WA, USA) and were implemented and calculated with Python. Logistic regression was performed using IBM SPSS Statistics (Version 2305 Build 16.0.16501.20074, Redmond, WA, USA).

## 3. Results

In the data from the German Burn Registry, several variables were listed. These can be found in Table 1. Firstly, we analyzed patient related data, which showed that more than 70% of patients are male and have an average age of 53 years. Concerning body measurements, patients showed average values of 1.75 m and a mean weight of 83 kg. Furthermore, attention was paid to 10 patient-related risk factors, which included obesity, smoking, and pre-existing illnesses such as diabetes mellitus, arterial hypertension, coronary artery disease, congestive heart failure, atrial fibrillation, renal failure, peripheral artery disease, and COPD. More than 20 percent of patients are smokers and around 20 percent show obesity. Other illnesses showed a range of 4.5 to 19.7 percent among all patients. Around two-thirds of all cases were primary admissions whereas one-third was transferred secondarily. Another interesting aspect is that over 60 percent of burn trauma happened in domestic surroundings, followed by occupational accidents (13.49%) and suicide (10.56%). Concerning the mechanism of trauma, more than half of the cases were due to flames followed by scalds as the second most common trauma cause. As one subform of scalds, burns due to fat are also captured. Hot fat reaches temperatures of 160 to 175 °C and therefore can lead to deeper burn wounds.

The mean average total burned surface area was 41.44% and the portion of full-thickness burns was calculated with a mean of 16.52%. Another interesting fact is that the mean temperature after admission was relatively low, under 36 °C. All in all, 32.48% of all patients died due to their burn injuries.

### 3.1. CART

**Figure 1:**
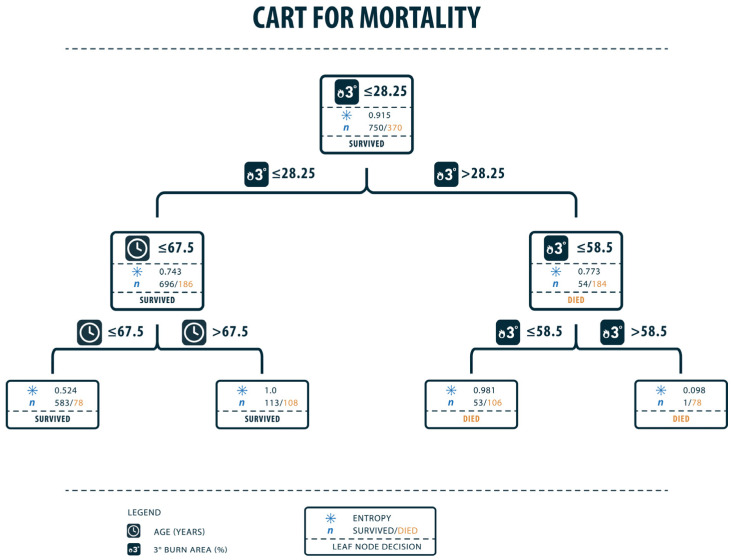
Classification and Regression Tree for mortality. Full-thickness burns on 28.25% of the body surface mark a point where it is more likely to die than to survive. In the left branch (patients who rather survive), the next differentiation is the patient’s age. Patients ≤ the age of 67.5 are more likely to survive. Furthermore, the right branch points out that full-thickness burns of >58.5% mean that patients die with almost absolute certainty. Training accuracy: 0.7938, Testing accuracy: 0.7363, Recall: 0.4888, F1-Score: 0.6027.

After analyzing the data from the German Burn registry with different training and testing splits, the results were unequivocal regarding one factor. Taking a training and testing split of 20/80%, full thickness burns make the most predictive factor for mortality, but not only the occurrence but especially the body surface area affected by full-thickness burns plays a significant role in mortality prediction. As shown in Figure 1, full-thickness burns of 28.25% represent the state where mortality rate is higher than survival (class 0 = survival: 882 patients; class 1 = mortality: 238 patients). In the cohort (class 1), full-thickness burns of 58.5% were the next factor in the following stage dividing patient population in survival and mortality. This points out the immense impact of full-thickness burns on patient mortality. Additionally, in the analysis of CARTs, the patient’s age was identified to be another significant factor in patient survival (age of 67.5 years: mortality rate is higher with 221 of 882 patients deceased). 

As a parameter for the quality of the splits, entropy was used in the CART analysis. The entropy is a measure which indicates the disorder of a feature and ranges from 0 (no disorder) to 1 (highest disorder).

### 3.2. Random Forest

For analysis of data via random forests, we took a training and testing split of 20/80%. This method of machine learning showed the patient’s age to be the most important factor for predicting mortality. Random forest demonstrated a median age of 71.5 years to be the turning point concerning the proportion of survival and mortality. In addition to this, full-thickness burns were identified as a significant factor predicting mortality (body surface area of 31.75% affected by full-thickness burns were the turning point).

### 3.3. XGBoost

By analyzing data from the German Burn registry with XGBoost via Python, we identified certain factors to have a significantly higher feature importance than others. The two most considerable factors are full-thickness burns (grade 3) with a feature importance score of 0.15 and the amount of total body surface area affected with an importance score of 0.1. Other noteworthy attributes are the patient’s age and the occurrence of an inhalation injury (importance score of 0.88 and 0.82). Training accuracy was 0.52 and testing accuracy was 0.344. 

### 3.4. Logistic Regression

Practicing logistic regression on the factor full-thickness burns and taking into consideration the regression coefficient of 0.052 leads to the conclusion that 1% TBSA of full-thickness burns increases the probability of mortality by about 5%.

Regarding the factor of age, the same procedure of logistic regression was performed leading to the result that one year of patient’s age leads to a higher mortality rate of 0.046, that is around 4%.

## 4. Discussion

Over the last decades a variety of scores for predicting survival in severely burned patients has been introduced. In the literature, several authors have compared different scores so far, coming to different conclusions regarding their accuracy. However, as a lot of innovations have revolutionized medicine over the last years, it seems obvious that prediction scores need further validation. 

In this study, we analyzed the data of severely burned patients with different mechanisms of machine learning. The results lead to the conclusion that full-thickness burns, the affected body surface, and patient’s age have the highest impact on mortality and therefore should be given the highest consideration when predicting the outcome after burn trauma. The results could be confirmed with each of the different methods of machine learning. 

**Figure 2:**
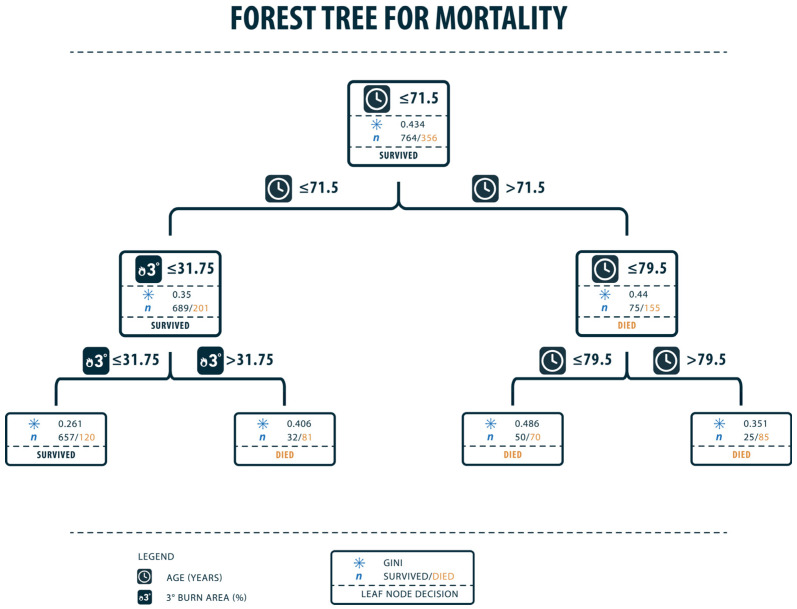
Forest tree for mortality. Patient’s age of 71.5 years marks a point where they are more likely to die than to survive. In the left branch (patients who rather survive), the next differentiation is regarding the occurrence of full-thickness burns on 31.75% of the body surface. Furthermore, the right branch points out that patient age of >79.5 years has the highest certainty for dying due to burns. The Gini Index was chosen as an indicator for impurity and ranges from 0 (lowest impurity) to 0.5 (highest impurity). Training accuracy: 0.7839, Testing accuracy: 0.7758, Recall: 0.3103, F1: 0.4615, Precision: 0.9.

CARTs and random forest trees put a focus on the most relevant factors concerning the prediction of mortality. After analyzing the data via Classification and Regression Trees, full-thickness burns of ≤28.5 percent predict a more likely chance of surviving the trauma of burns than >28.5 percent, as shown in Figure 1. Furthermore, when looking at the right branch of the CART, the extent of full-thickness burns predicts between the difference of rather not surviving and the almost absolute certainty of dying from the trauma. In this case, full-thickness burns of 58.5% mark the point where trauma in almost all cases leads to death and therefore marks an important factor for the prediction of mortality. In CART analysis, the entropy was quite high in the first split with 0.915. This makes sense as there is a lot of information and therefore high data variance. As 42 parameters and 1401 patients were analyzed, the entropy is high within the first splits. In random forest trees, patients’ age could be analyzed as another important predictive factor. In the first place, an age of >71.5 years could be pointed out as the point where the probability of dying is higher than surviving. Patients with an age of >79.5 years have an even higher probability of dying, so this marks another significant point for predicting mortality. In random forest trees, seen in Figure 2, the Gini Index as an indicator for impurity is quite high with a value of around 0.4. This again demonstrates the high variance within the data resulting from 42 different parameters in 1401 patients.

**Figure 3:**
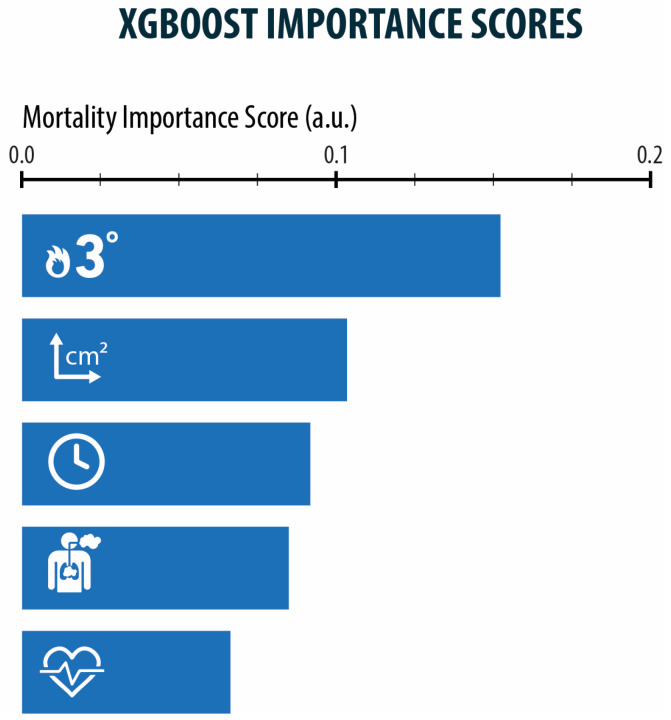
Depiction of the factors with the highest feature importance regarding mortality due to burns. In descending order, full-thickness burns, TBSA, patient’s age, inhalation injury, and cardiovascular comorbidities have the highest impact on the probability of mortality. Training accuracy: 0.5015, Testing accuracy: 0.4341, Recall: 0.23335, F1-Score: 0.6523033030.

**Figure 4:**
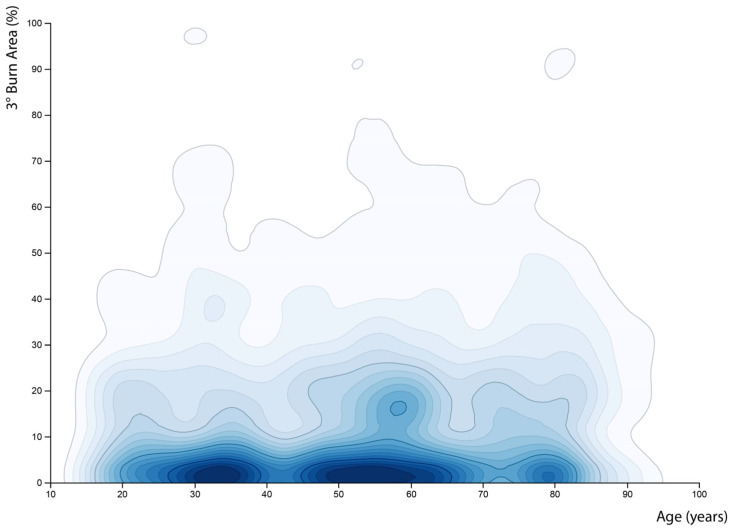
Contour plot for full-thickness burns and patients’ age. Correlation of the two factors was calculated via Kendall–Tau-b coefficient (0.058). This demonstrates that there is no correlation between these two factors, and patient’s age and degree of burn are independent factors for mortality.

Underlining these findings were the facts that 1% of full-thickness burns increases the probability of mortality by about 5% and one year of age increases it by about 4%. Figure 3 also demonstrates the factors full-thickness burns, TBSA and age as most relevant analysed via XGboost. These statements emphasize the impact of these factors on mortality and make it obvious that they should be given high consideration in predictive scores. On the other hand, the contour plot in Figure 4 also points out that the two mentioned factors do not correlate, therefore predictive scores should consider age and the extent of full-thickness burns separately. 

Comparing our findings to existing and established scores, it is noticeable that for example, in the ABSI score, relevant factors such as full-thickness burns or TBSA are included. Therefore, it is not surprising that studies have declared the ABSI to be still an accurate and valuable tool in predicting the mortality of severely burned patients [11]. In addition, recently published new scores such as the BUMP score include the variables that we identified as most significant [12]. However, existing scores clearly need some kind of revision as the variables mentioned are weighted evenly for calculations, as in the original ABSI score. Hence, methods of machine learning seem to be a rational method for weighting the impact of each factor and creating a variable. 

In the last years, artificial intelligence and especially machine learning have had a significant impact on diagnostics and therapy in medicine. For example, in the therapy of lung adenocarcinomas, multiple machine learning algorithms with optimal predictive performance were used to identify glutamine metabolism-related genes that can predict the effectiveness of certain immunotherapy [13]. Another impact of machine learning mechanisms is their usage regarding diagnostics, for example in the field of plastic surgery. A convolutional neuronal network-based system for instance could detect lung metastases in X-rays of patients with soft tissue sarcoma with an accuracy of 91.2% [14]. Moreover, according to the topic of risk scores and predictive models, machine learning already helped in generating novel prognostic models in the field of oncology. As an example, Shi et al. used six different machine learning mechanisms to identify genes correlated with hepatocellular carcinoma and generated a risk model using the mechanisms of artificial intelligence [15]. These cases demonstrate the wide range of applications for machine learning. 

The German Burn registry, as a platform that collects data from participating burn centers and therefore captures data from patients treated in intensive care units, is a suitable database for gathering relevant factors regarding complex injuries such as burns. However, as it was introduced only in 2014 and the occurrence of severe burn injuries is a rather rare trauma, the amount of data is still not sufficiently conclusive. Especially, machine learning algorithms such as XGBoost depend on a large amount of data to perform sufficient training and furthermore testing accuracy. Our analysis in XGboost showed full-thickness burns and the TBSA to be factors with the highest feature importance, but the training and testing accuracy were still low at 0.5 and 0.3. As the training accuracy was higher than the testing accuracy, this is an indicator for overfitting, which means that the machine learning correlates better with the training data than the test data. Furthermore, a testing accuracy of 0.3 is quite low which indicates a broad dataset. Working with a relatively small amount of data, this may put an emphasis on outliers in the dataset and therefore this might have an impact on the evaluation of a score. The models memorize the relatively small set of training data too well and therefore do not fit to the testing data as well. Mechanisms to avoid or minimize overfitting are k-fold cross-validation and larger datasets for training and feature selection. As we performed cross-validation in this study and also concentrated on the most relevant features for our study purpose, we assume that our model would need a significantly higher amount of data for performing a correct XGBoost analysis. Given the fact that burn trauma is a rather rare trauma, with cumulatively “only” 1401 patients within 5 years, but there is a huge set of parameters (42 in total) to analyze, it seems obvious that some parameters are outliers in the data. Therefore, collecting more data and therefore generating fewer outliers will be the best method to mitigate overfitting and thus improve the algorithm and prediction potential. 

Summarizing these facts, AI and especially machine learning represent a suitable and rational approach when analyzing large databases such as the German Burn Registry for creating a predictive score. However, for a valid analysis and subsequently creating a new, improved mortality prediction score for burn injuries, larger data volumes collected over more years would be beneficial. 

Another aspect that opens up during analysis of the mentioned parameters is the possibility of, for example, improving preclinical care. Considering only preclinical factors such as the temperature of the patients or the fact of primary or secondary admission or even the amount of administered fluid could therefore maybe show significant values which would emphasize the importance of certain factors in the preclinical setting. However, for these studies, larger datasets would also be necessary to be able to carry out valid analyses. 

Furthermore, machine learning could be used to analyze and improve clinical handling. Evaluating the data in Table 1, it seems controversial that there are more bronchoscopies performed than suspected inhalation injuries documented. Reasons for this might be certain standardized processes in some institutes which require a bronchoscopy for every ventilated patient after admission. Another reason might be the fact that cases with blistering dermal diseases are also included in the burn registry; therefore, the number of suspected inhalation injuries and accomplished bronchoscopies might differ. 

An additional interesting parameter is the fluid administration during the first days in the intensive care unit. The amount of administered fluid shows a wide range and has a high impact on the outcome of patients and can lead to certain complications [16]. The analysis of clinical data might also reveal interesting new insights into volume management regarding complications and might also be an interesting focus for usage of ML mechanisms in the future. 

Limitations of the study are, as mentioned before, the restricted number of patients involved. Especially in AI, more data lead to higher testing accuracy and therefore to better results. Another limitation is the limited number of chosen AI mechanisms; in the future, more and different mechanisms to evaluate these data may lead to further findings. 

## 5. Conclusions

During the last years, AI and machine learning have had a fundamental impact on different fields of medicine. Especially regarding predictive factors, machine learning provides benefits in analyzing relevant data. With the mechanisms of machine learning, we have been able to identify full-thickness burns, patient’s age, and TBSA as the most important factors regarding patient outcome in case of mortality. Concerning the different methods of machine learning, we identified especially XGboost to show overfitting. Therefore, one fundamental learning of this study was that we need larger datasets to generate sufficient accuracy. In the future, machine learning can be used to create a novel prediction score and analyze data from severely burned patients regarding other forms of outcome.

## Figures and Tables

**Table 1:** Patient population characteristics (*n* = 1401). The table gives an overview of patient-related factors, risk factors, the context and mechanism of the trauma, and factors concerning the trauma such as burned surface area (BSA), temperature, inhalation injury, and the number of deceased patients.

**Biological Gender**	*n*	%
- Male	1006	71.81%
- Female	395	28.19%
**Age (years)**	Mean	SD
	53	20
**Height**	Mean	SD
	1.75 m	0.09 m
**Weight**	Mean	SD
	83 kg	18 kg
**BMI**	Mean	SD
	26.82	5.48
**Risk Factors**	*n*	%
- Diabetes Mellitus	186	13.28%
- Peripheral Artery Disease	84	6.00%
- Coronary Artery Disease	172	12.28%
- Smoking	298	21.27%
- Arterial Hypertension	276	19.70%
- COPD	79	5.64%
- Atrial Fibrillation	91	6.50%
- Congestive Heart Disease	64	4.57%
- Obesity	272	19.41%
- Chronic Kidney Disease	142	10.14%
**Admission**	*n*	%
- Primary	964	68.81%
- Secondary	437	31.19%
**Context**	*n*	%
- Domestic	870	62.10%
- Occupation	189	13.49%
- Traffic	23	1.64%
- Suicide	148	10.56%
- Crime	35	2.50%
- Unknown	136	9.71%
**Mechanism**	*n*	%
- Scald	175	12.49%
- Flame	796	56.82%
- Fat	28	2.00%
- Contact Burn	15	1.0.7%
- Electricity	64	4.57%
- High Voltage	64	4.57%
- Explosion	114	8.14%
- Chemical	19	1.36%
- Unknown	126	8.99%
**BSA**	Mean	SD
- Superficial Dermal	13.59%	20.65%
- Deep Dermal	11.13%	12.33%
- Full Thickness	16.72%	21.84%
- TBSA	41.44%	21.67%
**Temperature (°C)**	Mean	SD
	35.92	1.36
**Suspected Inhalation Injury**	*n*	%
	489	34.90%
**Bronchoscopy**	*n*	%
	565	40.33%
**Verified Inhalation Injury**	*n*	%
	326	23.27%
**Deceased**	*n*	%
	455	32.48%
